# Supplementary material for: Neurovascular sequestration in paediatric P. falciparum malaria is visible clinically in the retina
Source: eLife. 2018 Mar 26;7:e32208. doi: 10.7554/eLife.32208 (PMC5898913; doi:10.7554/eLife.32208)
Supplement: Figure 4—source data 1. [file elife-32208-fig4-data1.docx]

**Figure 4 – Source file 1.**

| Case # | MR grade | CD34 Cap | | CD34 Ven | | SMAVen | | PDGFRb Cap | | PDGFRb Ven | |
| --- | --- | --- | --- | --- | --- | --- | --- | --- | --- | --- | --- |
|  |  | pRBC | npRBC | pRBC | npRBC | pRBC | npRBC | pRBC | npRBC | pRBC | npRBC |
| 1 | 2 | 31 | -- | 6 | -- | 3 | -- | 23 | -- | 7 | -- |
| 2 | 2 | 2 | -- | 3 | -- | 12 | -- | 0 | -- | 0 | -- |
| 3 | 2 | 0 | -- | 0 | -- | 6 | -- | 10 | -- | 18 | -- |
| 4 | 2 | 14 | -- | 9 | -- | 14 | -- | 3 | -- | 0 | -- |
| 5 | 2 | n/a | -- | n/a | -- | 5 | -- | 36 | -- | 41 | -- |
| 6 | 2 | n/a | -- | n/a | -- | 0 | -- | 70 | -- | 31 | -- |
| 7 | 2 | n/a | -- | n/a | -- | 2 | -- | 12 | -- | 7 | -- |
| 8 | 2 | n/a | -- | n/a | -- | 15 | -- | 53 | -- | 21 | -- |
| 9 | 2 | n/a | -- | n/a | -- | 0 | -- | 27 | -- | 16 | -- |
| 10 | 2 | n/a | -- | n/a | -- | 0 | -- | 0 | -- | 0 | -- |
| 11 | 2 | 0 | -- | 5 | -- | 0 | -- | 48 | -- | 36 | -- |
| 12 | 2 | 3 | -- | 0 | -- | 28 | -- | 5 | -- | 5 | -- |
| 13 | 2 | 7 | -- | 10 | -- | 20 | -- | 14 | -- | 20 | -- |
| 14 | 2 | 0 | -- | 13 | -- | 14 | -- | 22 | -- | 22 | -- |
| 15 | 1 | n/a | -- | n/a | -- | n/a | -- | 21 | -- | 18 | -- |
| 16 | 2 | 0 | -- | 8 | -- | 10 | -- | 20 | -- | 25 | -- |
| 17 | 1 | 0 | 86 | 0 | 65 | 15 | 61 | 0 | 64 | 15 | 75 |
| 19 | 1 | n/a | 100 | 55.5 | 100 | 7 | 81 | 27 | 56 | 17 | 50 |
| 20 | 1 | n/a | n/a | n/a | n/a | n/a | n/a | 15 | n/a | 22 | n/a |
| 21 | 1 | 43 | 100 | 10 | 97 | 15 | 75 | 19 | 37 | 13 | 50 |
| 22 | 1 | 7 | 90.3 | 10 | 90.6 | 34 | 68 | 14 | 57 | 23 | 100 |
| 23 | 0 | -- | n/a | -- | n/a | -- | 77 | -- | 98 | -- | 92 |
| 24 | 0 | -- | 86 | -- | 86 | -- | 86 | -- | 82 | -- | 70 |
| 25 | 0 | -- | n/a | -- | n/a | -- | 49 | -- | 100 | -- | 89 |
| 26 | 0 | -- | 100 | -- | 73 | -- | 59 | -- | 93 | -- | 93 |
| 27 | 0 | -- | 84 | -- | 88 | -- | 71 | -- | 82 | -- | 93 |
| 28 | 0 | -- | 98 | -- | 93 | -- | 88 | -- | 75 | -- | 71 |
| 29 | 0 | -- | n/a | -- | n/a | -- | n/a | -- | 82 | -- | 69 |

MR grade=malarial retinopathy grade. Cap=% capillaries with continuous marker staining; Ven=% venules with continuous marker staining. pRBC=presence of parasitised red blood cells sequestration in the vessel analysed; npRBC= absence of parasitised red blood cells sequestration in the vessel analysed. n/a= not available. -- = analysis not applicable.
