# Supplementary material for: Neurovascular sequestration in paediatric P. falciparum malaria is visible clinically in the retina
Source: eLife. 2018 Mar 26;7:e32208. doi: 10.7554/eLife.32208 (PMC5898913; doi:10.7554/eLife.32208)
Supplement: Figure 6—source data 1. [file elife-32208-fig6-data1.docx]

**Figure 6 – Source file 1.**

| Case # | MR grade | Macular whitening | VEGFR1 | | | |
| --- | --- | --- | --- | --- | --- | --- |
|  |  |  | GCL | IPL | INL | OPL |
| 1 | 2 | 1/3-1 DA | 13 | 31 | 12 | 24 |
| 2 | 2 | ≥1 DA | 17 | 43 | 16 | 29 |
| 3 | 2 | ≥1 DA | 25 | 38 | 15 | 36 |
| 4 | 2 | <1/3 DA | 10 | 8 | 2 | 7 |
| 5 | 2 | ≥1 DA | n/a | n/a | n/a | n/a |
| 6 | 2 | ≥1 DA | 24 | 29 | 19 | 11 |
| 7 | 2 | ≥1 DA | 19 | 18 | 16 | 13 |
| 8 | 2 | ≥1 DA | 14 | 17 | 9 | 16 |
| 9 | 2 | ≥1 DA | 19 | 38 | 22 | 28 |
| 10 | 2 | 1/3-1 DA | 17 | 29 | 11 | 22 |
| 11 | 2 | 1/3-1 DA | 15 | 30 | 12 | 23 |
| 12 | 2 | 1/3-1 DA | 14 | 29 | 11 | 24 |
| 13 | 2 | ≥1 DA | 17 | 33 | 18 | 18 |
| 14 | 2 | <1/3 DA | 9 | 6 | 3 | 13 |
| 15 | 1 | <1/3 DA | n/a | n/a | n/a | n/a |
| 16 | 2 | <1/3 DA | 7 | 11 | 2 | 11 |
| 17 | 1 | <1/3 DA | 13 | 14 | 11 | 11 |
| 19 | 1 | <1/3 DA | 9 | 21 | 12 | 17 |
| 20 | 1 | <1/3 DA | 7 | 12 | 6 | 14 |
| 21 | 1 | <1/3 DA | 7 | 11 | 3 | 8 |
| 22 | 1 | None | 10 | 20 | 11 | 20 |
| 23 | 0 | None | 2 | 3 | 1 | 0 |
| 24 | 0 | None | 4 | 12 | 10 | 6 |
| 25 | 0 | None | 3 | 14 | 2 | 11 |
| 26 | 0 | None | 5 | 13 | 4 | 10 |
| 27 | 0 | None | 7 | 24 | 12 | 24 |
| 28 | 0 | None | 4 | 12 | 6 | 12 |
| 29 | 0 | None | 4 | 7 | 11 | 11 |

MR grade=malarial retinopathy grade. VEGR1: intensity of VEGFR1 staining measured in the retinal layer. GCL: ganglion cells layer; IPL: inner plexiform layer; INL: inner nuclear layer; OPL: outer plexiform layer. n/a= not available.
