# Supplementary material for: Neurovascular sequestration in paediatric P. falciparum malaria is visible clinically in the retina
Source: eLife. 2018 Mar 26;7:e32208. doi: 10.7554/eLife.32208 (PMC5898913; doi:10.7554/eLife.32208)
Supplement: Figure 7—source data 1. [file elife-32208-fig7-data1.docx]

**Figure 7 – Source file 1.**

| Case # | MR grade | Macular whitening | AQP4 | | | |
| --- | --- | --- | --- | --- | --- | --- |
|  |  |  | NFL | GCL | IPL | OPL |
| 1 | 2 | 1/3-1 DA | 31 | 32 | 22 | 20 |
| 2 | 2 | ≥1 DA | 102 | 44 | 20 | 70 |
| 3 | 2 | ≥1 DA | 40 | 44 | 10 | 77 |
| 4 | 2 | <1/3 DA | 28 | 32 | 14 | 25 |
| 5 | 2 | ≥1 DA | n/a | n/a | n/a | n/a |
| 6 | 2 | ≥1 DA | 56 | 39 | 26 | 40 |
| 7 | 2 | ≥1 DA | 52 | 42 | 10 | 51 |
| 8 | 2 | ≥1 DA | 72 | 46 | 21 | 97 |
| 9 | 2 | ≥1 DA | 53 | 41 | 8 | 76 |
| 10 | 2 | 1/3-1 DA | 42 | 37 | 15 | 43 |
| 11 | 2 | 1/3-1 DA | 37 | 33 | 16 | 50 |
| 12 | 2 | 1/3-1 DA | 46 | 33 | 12 | 35 |
| 13 | 2 | ≥1 DA | 48 | 67 | 7 | 103 |
| 14 | 2 | <1/3 DA | 19 | 24 | 15 | 25 |
| 15 | 1 | <1/3 DA | n/a | n/a | n/a | n/a |
| 16 | 2 | <1/3 DA | 25 | 28 | 14 | 25 |
| 17 | 1 | <1/3 DA | 44 | 41 | 23 | 52 |
| 19 | 1 | <1/3 DA | 44 | 32 | 17 | 61 |
| 20 | 1 | <1/3 DA | 25 | 38 | 11 | 26 |
| 21 | 1 | <1/3 DA | 76 | 58 | 30 | 60 |
| 22 | 1 | None | 50 | 62 | 39 | 84 |
| 23 | 0 | None | 24 | 22 | 12 | 9 |
| 24 | 0 | None | 27 | 13 | 7 | 16 |
| 25 | 0 | None | 20 | 15 | 9 | 12 |
| 26 | 0 | None | 25 | 17 | 5 | 11 |
| 27 | 0 | None | 39 | 47 | 15 | 43 |
| 28 | 0 | None | 19 | 18 | 8 | 12 |
| 29 | 0 | None | 14 | 12 | 6 | 10 |

MR grade=malarial retinopathy grade. AQP4 intensity of AQP4 staining measured in the retinal layer. NFL: nerve fibre layer; GCL: ganglion cells layer; IPL: inner plexiform layer; OPL: outer plexiform layer. n/a= specimen not available.
