# Supplementary material for: Neurovascular sequestration in paediatric P. falciparum malaria is visible clinically in the retina
Source: eLife. 2018 Mar 26;7:e32208. doi: 10.7554/eLife.32208 (PMC5898913; doi:10.7554/eLife.32208)
Supplement: Figure 7—figure supplement 1—source data 1. [file elife-32208-fig7-figsupp1-data1.docx]

**Figure 7 Supplement 1 – Source file 1.**

| Case # | MR grade | Temporal peripheral whitening | AQP4 | | | |
| --- | --- | --- | --- | --- | --- | --- |
|  |  |  | NFL | GCL | IPL | OPL |
| 1 | 2 | 3 | 30 | n/a | 10 | 20 |
| 2 | 2 | 3 | 126 | n/a | 62 | 60 |
| 3 | 2 | 1 | 131 | n/a | 59 | 90 |
| 4 | 2 | 2 | 44 | n/a | 9 | 21 |
| 5 | 2 | 3 | n/a | n/a | n/a | n/a |
| 6 | 2 | 2 | 62 | n/a | 19 | 52 |
| 7 | 2 | 1 | 124 | n/a | 75 | 110 |
| 8 | 2 | 2 | 92 | n/a | 50 | 59 |
| 9 | 2 | 1 | 64 | n/a | 22 | 31 |
| 10 | 2 | 2 | 116 | n/a | 63 | 68 |
| 11 | 2 | 2 | 100 | n/a | 56 | 60 |
| 12 | 2 | 0 | 76 | n/a | 38 | 59 |
| 13 | 2 | 1 | 124 | n/a | 73 | 115 |
| 14 | 2 | n/a | 53 | n/a | 33 | 40 |
| 15 | 1 | 1 | n/a | n/a | n/a | n/a |
| 16 | 2 | 0 | 76 | n/a | 36 | 54 |
| 17 | 1 | 1 | 43 | n/a | 26 | 39 |
| 19 | 1 | 1 | 65 | n/a | 25 | 39 |
| 20 | 1 | 1 | 64 | n/a | 29 | 40 |
| 21 | 1 | n/a | 64 | n/a | 38 | 29 |
| 22 | 1 | None | 126 | n/a | 93 | 116 |
| 23 | 0 | None | 28 | 38 | 27 | 26 |
| 24 | 0 | None | 53 | 42 | 26 | 37 |
| 25 | 0 | None | 11 | n/a | 11 | 14 |
| 26 | 0 | None | 15 | 45 | 14 | 12 |
| 27 | 0 | None | 20 | n/a | 44 | 54 |
| 28 | 0 | None | 19 | 39 | 17 | 44 |
| 29 | 0 | None | 30 | n/a | 20 | 16 |

MR grade=malarial retinopathy grade. AQP4 intensity of AQP4 staining measured in the retinal layer. NFL: nerve fibre layer; GCL: ganglion cells layer; IPL: inner plexiform layer; OPL: outer plexiform layer. n/a= not available.
