# Supplementary material for: Neurovascular sequestration in paediatric P. falciparum malaria is visible clinically in the retina
Source: eLife. 2018 Mar 26;7:e32208. doi: 10.7554/eLife.32208 (PMC5898913; doi:10.7554/eLife.32208)
Supplement: Figure 9—source data 1. [file elife-32208-fig9-data1.docx]

**Barrera MacCormick et al Figure 9 -source data 1**

Frequency of visible sequestration on fluorescein angiography in 6 microvessel types in 259 subjects with retinopathy positive CM and the odds ratios of death within the admission

Intravascular filling defects on fluorescein angiography

| Vessel type | present | absent | cannot grade | present (%) | association with death | | | |
| --- | --- | --- | --- | --- | --- | --- | --- | --- |
|  |  |  |  |  | odds | 95% confidence limits | | p |
| Large venules | 173 | 69 | 17 | 66.8 | 1.42 | 0.52 | 3.9 | 0.49 |
| Small venules | 224 | 17 | 18 | 86.5 | 0.55 | 0.06 | 5.09 | 0.6 |
| Post-capillary venules | 198 | 7 | 54 | 76.4 | 0.37 | 0.04 | 3.7 | 0.4 |
| Pre-capillary arterioles | 38 | 61 | 160 | 36.7 | 2.47 | 0.94 | 6.45 | 0.06 |
| Small arterioles | 95 | 99 | 65 | 29.7 | 1.43 | 0.66 | 3.11 | 0.37 |
| Large arterioles | 77 | 161 | 21 | 10.4 | 2.81 | 1.17 | 6.72 | 0.02 |
