# Supplementary material for: Neurovascular sequestration in paediatric P. falciparum malaria is visible clinically in the retina
Source: eLife. 2018 Mar 26;7:e32208. doi: 10.7554/eLife.32208 (PMC5898913; doi:10.7554/eLife.32208)
Supplement: Figure 10—source data 1. [file elife-32208-fig10-data1.docx]

**Barrera MacCormick et al Figure 10 -source data 1**

Proportion of vessel detected by semiautomated quantitative analysis of sequestration related to survival in 251 eyes (1 eye per case).

| Subject | Ratio affected:unaffected |  | Rank | Died |
| --- | --- | --- | --- | --- |
| 2402 | 0.11 |  | 1 |  |
| 2547 | 0.12 |  | 2 |  |
| 2898 | 0.13 |  | 3 |  |
| 2466 | 0.13 |  | 4 |  |
| 2632 | 0.13 |  | 5 |  |
| 2633 | 0.15 |  | 6 |  |
| 2752 | 0.18 |  | 7 | died |
| 2694 | 0.18 |  | 8 |  |
| 2952 | 0.18 |  | 9 | died |
| 2749 | 0.18 |  | 10 |  |
| 2635 | 0.18 |  | 11 |  |
| 2778 | 0.19 |  | 12 |  |
| 2820 | 0.20 |  | 13 |  |
| 3140 | 0.20 |  | 14 | died |
| 3264 | 0.20 |  | 15 |  |
| 2492 | 0.21 |  | 16 |  |
| 2503 | 0.21 |  | 17 |  |
| 2742 | 0.21 |  | 18 |  |
| 3173 | 0.21 |  | 19 |  |
| 3236 | 0.22 |  | 20 |  |
| 2650 | 0.22 |  | 21 |  |
| 2377 | 0.22 |  | 22 |  |
| 2724 | 0.23 |  | 23 |  |
| 3183 | 0.23 |  | 24 |  |
| 3234 | 0.24 |  | 25 |  |
| 2740 | 0.24 |  | 26 |  |
| 3245 | 0.24 |  | 27 |  |
| 3266 | 0.24 |  | 28 |  |
| 2672 | 0.25 |  | 29 |  |
| 3242 | 0.25 |  | 30 |  |
| 3137 | 0.25 |  | 31 |  |
| 2666 | 0.26 |  | 32 |  |
| 2502 | 0.26 |  | 33 |  |
| 2673 | 0.26 |  | 34 |  |
| 2836 | 0.26 |  | 35 |  |
| 2551 | 0.26 |  | 36 |  |
| 2923 | 0.26 |  | 37 |  |
| 2550 | 0.26 |  | 38 |  |
| 2531 | 0.27 |  | 39 |  |
| 2644 | 0.27 |  | 40 |  |
| 2536 | 0.27 |  | 41 |  |
| 2781 | 0.27 |  | 42 |  |
| 2794 | 0.27 |  | 43 |  |
| 2892 | 0.27 |  | 44 |  |
| 3136 | 0.27 |  | 45 |  |
| 2743 | 0.27 |  | 46 |  |
| 2767 | 0.28 |  | 47 |  |
| 2802 | 0.28 |  | 48 | died |
| 3170 | 0.28 |  | 49 |  |
| 3108 | 0.28 |  | 50 |  |
| 2544 | 0.28 |  | 51 |  |
| 2522 | 0.28 |  | 52 |  |
| 2636 | 0.28 |  | 53 |  |
| 2479 | 0.29 |  | 54 |  |
| 2727 | 0.29 |  | 55 |  |
| 2546 | 0.29 |  | 56 |  |
| 2662 | 0.29 |  | 57 |  |
| 3194 | 0.30 |  | 58 |  |
| 2800 | 0.30 |  | 59 |  |
| 2353 | 0.30 |  | 60 |  |
| 2777 | 0.30 |  | 61 |  |
| 3251 | 0.30 |  | 62 |  |
| 2476 | 0.30 |  | 63 |  |
| 2526 | 0.30 |  | 64 |  |
| 3261 | 0.31 |  | 65 |  |
| 3243 | 0.31 |  | 66 |  |
| 3162 | 0.31 |  | 67 | died |
| 3176 | 0.31 |  | 68 |  |
| 2540 | 0.31 |  | 69 |  |
| 2657 | 0.31 |  | 70 |  |
| 2655 | 0.31 |  | 71 |  |
| 2352 | 0.31 |  | 72 |  |
| 2640 | 0.32 |  | 73 | died |
| 2444 | 0.32 |  | 74 |  |
| 2652 | 0.32 |  | 75 |  |
| 2578 | 0.32 |  | 76 |  |
| 2700 | 0.32 |  | 77 |  |
| 2607 | 0.32 |  | 78 |  |
| 2670 | 0.32 |  | 79 |  |
| 2565 | 0.32 |  | 80 |  |
| 2871 | 0.33 |  | 81 | died |
| 2521 | 0.33 |  | 82 |  |
| 2691 | 0.33 |  | 83 |  |
| 3231 | 0.33 |  | 84 |  |
| 3280 | 0.33 |  | 85 |  |
| 3200 | 0.33 |  | 86 |  |
| 3254 | 0.33 |  | 87 |  |
| 3220 | 0.33 |  | 88 |  |
| 2387 | 0.33 |  | 89 |  |
| 2448 | 0.33 |  | 90 |  |
| 3177 | 0.33 |  | 91 |  |
| 2788 | 0.33 |  | 92 |  |
| 2566 | 0.33 |  | 93 |  |
| 2576 | 0.34 |  | 94 |  |
| 2570 | 0.34 |  | 95 |  |
| 3256 | 0.34 |  | 96 |  |
| 2553 | 0.34 |  | 97 |  |
| 2359 | 0.34 |  | 98 |  |
| 3189 | 0.34 |  | 99 |  |
| 2704 | 0.34 |  | 100 | died |
| 2568 | 0.34 |  | 101 |  |
| 3193 | 0.34 |  | 102 |  |
| 2667 | 0.34 |  | 103 |  |
| 2857 | 0.34 |  | 104 |  |
| 3150 | 0.34 |  | 105 |  |
| 3131 | 0.34 |  | 106 |  |
| 2610 | 0.34 |  | 107 |  |
| 3238 | 0.34 |  | 108 |  |
| 2360 | 0.35 |  | 109 |  |
| 3271 | 0.35 |  | 110 |  |
| 2373 | 0.35 |  | 111 |  |
| 3217 | 0.35 |  | 112 |  |
| 2819 | 0.35 |  | 113 |  |
| 2516 | 0.35 |  | 114 |  |
| 2411 | 0.35 |  | 115 |  |
| 2594 | 0.35 |  | 116 |  |
| 2763 | 0.35 |  | 117 | died |
| 2539 | 0.35 |  | 118 |  |
| 3112 | 0.35 |  | 119 |  |
| 3124 | 0.35 |  | 120 |  |
| 2649 | 0.35 |  | 121 |  |
| 2645 | 0.35 |  | 122 |  |
| 2885 | 0.36 |  | 123 | died |
| 2481 | 0.36 |  | 124 |  |
| 3214 | 0.36 |  | 125 |  |
| 3225 | 0.36 |  | 126 | died |
| 2850 | 0.36 |  | 127 | died |
| 2877 | 0.36 |  | 128 | died |
| 3270 | 0.37 |  | 129 |  |
| 3172 | 0.37 |  | 130 |  |
| 2944 | 0.37 |  | 131 |  |
| 3156 | 0.37 |  | 132 |  |
| 2626 | 0.37 |  | 133 | died |
| 3154 | 0.37 |  | 134 |  |
| 3221 | 0.37 |  | 135 |  |
| 3110 | 0.37 |  | 136 |  |
| 2450 | 0.37 |  | 137 |  |
| 3253 | 0.37 |  | 138 | died |
| 3133 | 0.38 |  | 139 |  |
| 2617 | 0.38 |  | 140 | died |
| 2629 | 0.38 |  | 141 |  |
| 3155 | 0.38 |  | 142 |  |
| 3288 | 0.38 |  | 143 |  |
| 3226 | 0.38 |  | 144 |  |
| 3206 | 0.38 |  | 145 |  |
| 3218 | 0.38 |  | 146 |  |
| 3258 | 0.38 |  | 147 | died |
| 2510 | 0.38 |  | 148 |  |
| 2948 | 0.39 |  | 149 |  |
| 3250 | 0.40 |  | 150 |  |
| 2867 | 0.40 |  | 151 |  |
| 2913 | 0.40 |  | 152 |  |
| 3132 | 0.40 |  | 153 |  |
| 2623 | 0.40 |  | 154 | died |
| 3287 | 0.40 |  | 155 |  |
| 2941 | 0.41 |  | 156 |  |
| 3241 | 0.41 |  | 157 |  |
| 2669 | 0.41 |  | 158 |  |
| 2886 | 0.41 |  | 159 |  |
| 2864 | 0.41 |  | 160 |  |
| 3192 | 0.41 |  | 161 |  |
| 3255 | 0.41 |  | 162 |  |
| 3196 | 0.41 |  | 163 |  |
| 3106 | 0.41 |  | 164 |  |
| 2903 | 0.42 |  | 165 |  |
| 3142 | 0.42 |  | 166 |  |
| 2647 | 0.42 |  | 167 |  |
| 3216 | 0.42 |  | 168 |  |
| 2619 | 0.43 |  | 169 |  |
| 3207 | 0.43 |  | 170 |  |
| 2887 | 0.44 |  | 171 |  |
| 3185 | 0.44 |  | 172 |  |
| 3227 | 0.44 |  | 173 |  |
| 3232 | 0.44 |  | 174 |  |
| 3203 | 0.44 |  | 175 |  |
| 3273 | 0.44 |  | 176 |  |
| 2449 | 0.45 |  | 177 |  |
| 2703 | 0.45 |  | 178 |  |
| 2432 | 0.45 |  | 179 |  |
| 2929 | 0.45 |  | 180 |  |
| 3198 | 0.45 |  | 181 |  |
| 3186 | 0.45 |  | 182 |  |
| 2507 | 0.45 |  | 183 | died |
| 2509 | 0.46 |  | 184 |  |
| 3116 | 0.46 |  | 185 | died |
| 2878 | 0.46 |  | 186 | died |
| 3178 | 0.46 |  | 187 | died |
| 3103 | 0.46 |  | 188 |  |
| 3187 | 0.46 |  | 189 |  |
| 2400 | 0.46 |  | 190 |  |
| 2968 | 0.47 |  | 191 |  |
| 3278 | 0.47 |  | 192 |  |
| 3149 | 0.47 |  | 193 |  |
| 2382 | 0.47 |  | 194 |  |
| 2631 | 0.47 |  | 195 |  |
| 3246 | 0.47 |  | 196 |  |
| 3224 | 0.47 |  | 197 |  |
| 3101 | 0.47 |  | 198 |  |
| 2350 | 0.47 |  | 199 |  |
| 3228 | 0.48 |  | 200 |  |
| 3182 | 0.48 |  | 201 |  |
| 2475 | 0.48 |  | 202 |  |
| 3128 | 0.48 |  | 203 |  |
| 3235 | 0.48 |  | 204 | died |
| 2527 | 0.48 |  | 205 |  |
| 2902 | 0.49 |  | 206 |  |
| 3209 | 0.49 |  | 207 |  |
| 2940 | 0.49 |  | 208 |  |
| 3212 | 0.49 |  | 209 | died |
| 2692 | 0.50 |  | 210 |  |
| 2609 | 0.50 |  | 211 |  |
| 3129 | 0.51 |  | 212 |  |
| 2342 | 0.52 |  | 213 |  |
| 2351 | 0.52 |  | 214 | died |
| 3286 | 0.53 |  | 215 | died |
| 3290 | 0.53 |  | 216 |  |
| 2932 | 0.54 |  | 217 |  |
| 2603 | 0.54 |  | 218 | died |
| 2616 | 0.54 |  | 219 |  |
| 3223 | 0.54 |  | 220 |  |
| 2653 | 0.55 |  | 221 | died |
| 3120 | 0.56 |  | 222 | died |
| 2771 | 0.56 |  | 223 | died |
| 3169 | 0.56 |  | 224 |  |
| 3127 | 0.57 |  | 225 |  |
| 2383 | 0.57 |  | 226 |  |
| 3275 | 0.57 |  | 227 |  |
| 3222 | 0.57 |  | 228 |  |
| 2907 | 0.57 |  | 229 |  |
| 3284 | 0.58 |  | 230 |  |
| 3281 | 0.58 |  | 231 |  |
| 2601 | 0.58 |  | 232 | died |
| 3279 | 0.58 |  | 233 |  |
| 2438 | 0.60 |  | 234 |  |
| 2624 | 0.60 |  | 235 |  |
| 3157 | 0.61 |  | 236 |  |
| 3248 | 0.61 |  | 237 |  |
| 3229 | 0.62 |  | 238 |  |
| 3289 | 0.62 |  | 239 |  |
| 3283 | 0.63 |  | 240 |  |
| 2723 | 0.65 |  | 241 | died |
| 3260 | 0.65 |  | 242 |  |
| 3230 | 0.65 |  | 243 |  |
| 3237 | 0.65 |  | 244 |  |
| 2345 | 0.65 |  | 245 |  |
| 2675 | 0.65 |  | 246 |  |
| 3115 | 0.66 |  | 247 |  |
| 3269 | 0.67 |  | 248 |  |
| 2922 | 0.67 |  | 249 |  |
| 2404 | 0.76 |  | 250 | died |
| 3268 | 0.77 |  | 251 |  |
|  |  |  |  |  |
| Totals |  |  | 251 | 33 |
