# Supplementary material for: Neurovascular sequestration in paediatric P. falciparum malaria is visible clinically in the retina
Source: eLife. 2018 Mar 26;7:e32208. doi: 10.7554/eLife.32208 (PMC5898913; doi:10.7554/eLife.32208)
Supplement: Supplementary file 1. [file elife-32208-supp1.docx]

## Supplementary file 1

Comparison of children without (515) and with (1160) admission retinal exam data. Groups were compared using univariate logistic regression. Values of p are highlighted if ≤ 0.01. *p-value calculated by Kruskal-Wallis test instead of logistic regression.

| Variable name | Units | Children without admission eye exam | | | Children with admission eye exam | | | Odds of no retinal exam | | |  |  |
| --- | --- | --- | --- | --- | --- | --- | --- | --- | --- | --- | --- | --- |
|  |  | Numerical characteristics | | n | Numerical characteristics | | n | OR | 95%CI | p |  |  |
| Demographics | | | | | | | | | | |  |  |
| Age  (median, IQR) | months | 34 | 22-55 | 514 | 39 | 25-60 | 1158 | 1.01 | 1.00-1.01 | **<0.001** |  |  |
| Weight  (median, IQR) | kg | 11.8 | 9.4-14.2 | 515 | 12 | 10-15 | 1160 | 1.03 | 1.01-1.06 | **0.005** |  |  |
| Height  (median, IQR) | cm | 89 | 78-100 | 503 | 91 | 81-103 | 1141 | 1.01 | 1.00-1.02 | **0.001** |  |  |
| Sex  (%) | boy | 49.7 |  | 256 | 48.7 |  | 563 |  |  |  |  |  |
|  | girl | 50.3 |  | 259 | 51.3 |  | 594 | 1.04 | 0.85-1.28 | 0.69 |  |  |
| Clinical | | | | | | | | | | |  |  |
| Coma score  (%) | 0 | 16.1 |  | 83 | 12.7 |  | 148 |  |  |  |  |  |
|  | 1 | 42.9 |  | 221 | 39.7 |  | 461 | 1.17 | 0.86-1.60 | 0.37 |  |  |
|  | 2 | 41.0 |  | 211 | 47.5 |  | 551 | 1.46 | 1.07-2.00 | 0.017 |  |  |
| Respiratory distress (%) | absent | 58.5 |  | 299 | 61.6 |  | 714 |  |  |  |  |  |
|  | present | 41.5 |  | 212 | 38.4 |  | 445 | 0.88 | 0.71-1.09 | 0.23 |  |  |
| Convulsions at admission (%) | absent | 83.5 |  | 429 | 82.9 |  | 954 |  |  |  |  |  |
|  | present | 16.5 |  | 85 | 17.1 |  | 197 | 1.04 | 0.79-1.38 | 0.78 |  |  |
| Laboratory | | | | | | | | | | |  |  |
| Parasitaemia (median, IQR) | #cells | 78396 | 15330-227000 | 500 | 68012 | 11250-278000 | 1108 | 1 | 0.99-1.00 | 0.10 |  |  |
| White cell count (median, IQR) | #cells | 9800 | 6800-15025 | 470 | 9700 | 6800-14300 | 1058 | 0.99 | 0.99-1.00 | 0.69 |  |  |
| Haematocrit  (median, IQR) | % | 22 | 16-28 | 511 | 22 | 17-28 | 1150 | 1 | 0.99-1.02 | 0.50 |  |  |
| Lactate  (median, IQR) | mmol/L | 7 | 3.4-12.1 | 321 | 5.6 | 3.2-9.4 | 821 | 0.95 | 0.92-0.98 | **<0.001** |  |  |
| HRP2  (median, IQR) | ng/ml | 6915 | 3312-12102 | 152 | 5855 | 2360-10434 | 774 | 0.99 | 0.99-1.00 | 0.06 |  |  |
| HIV  (%) | negative | 84.3 |  | 360 | 84.5 |  | 850 |  |  |  |  |  |
|  | positive | 15.7 |  | 67 | 15.5 |  | 156 | 0.99 | 0.72-1.35 | 0.93 |  |  |
| Outcomes | | | | | | | | | | |  |  |
| Recovery  status (%) | full recovery | 68.5 |  | 353 | 75.7 |  | 878 |  |  |  |  |  |
|  | sequelae | 10.7 |  | 55 | 8.5 |  | 99 | 0.72 | 0.51-1.03 | 0.07 |  |  |
|  | died | 20.8 |  | 107 | 15.8 |  | 183 | 0.69 | 0.53-0.90 | **0.006** |  |  |
| Time to consciousness  (median, IQR) | hours | 16 | 8-40 | 396 | 16 | 8-36 | 952 | 0.99 | 0.99-1.00 | 0.75 |  |  |
| Time to death (median, IQR) | hours | 8 | 3-24 | 107 | 17 | 6-31 | 183 |  |  | **0.001*** |  |  |
